# Early Osteogenic Transcriptional Response of Pre-Osteoblastic Cells on Biomorphic Mg^2+^-Doped Biphasic Calcium Phosphate Scaffolds

**DOI:** 10.3390/cells15201885

**Published:** 2026-10-15

**Authors:** Francesco Pansera, Alberto Ballardini, Giacomo Morozzi, Alice Pasini, Emanuele Giordano

**Affiliations:** 1 Laboratory of Cellular and Molecular Engineering “S. Cavalcanti”, Department of Electrical, Electronic and Information Engineering “G. Marconi” (DEI), University of Bologna, 47521 Cesena, Italy; alice.pasini@unibo.it (A.P.); emanuele.giordano@unibo.it (E.G.); 2 GreenBone Ortho S.p.A., 48018 Faenza, Italy; alberto.ballardini@greenbone.it (A.B.); giacomo.morozzi@studio.unibo.it (G.M.); 3 Advanced Research Center on Electronic Systems (ARCES), University of Bologna, 40125 Bologna, Italy

**Keywords:** biomorphic scaffolds, biphasic calcium phosphate, bone tissue engineering, hydroxyapatite, magnesium substitution, MC3T3-E1, osteogenic commitment, β-tricalcium phosphate

## Abstract

Critical-sized bone defects remain a major clinical challenge because of their limited regenerative capacity and the high incidence of delayed healing and non-union. Biomorphic calcium phosphate scaffolds have emerged as promising bone tissue engineering (BTE) platforms owing to their ability to reproduce the hierarchical architecture of trabecular bone while providing a bioactive environment for bone regeneration. In the present study, the early osteogenic transcriptional response of biomorphic magnesium-doped hydroxyapatite (HA)/β-tricalcium phosphate (β-TCP) scaffolds obtained through biomorphic transformation of a rattan wood template was investigated to evaluate their ability to support the early osteogenic response of pre-osteoblastic cells. Porous scaffolds preserving the hierarchical architecture of the rattan template and exhibiting different HA/β-TCP phase compositions were obtained through a multistep biomorphic transformation process followed by controlled magnesium incorporation. Cytocompatibility was assessed using MC3T3-E1 pre-osteoblastic cells by evaluating cell viability, adhesion and proliferation. The early osteogenic response was investigated by analyzing the expression of representative osteogenic marker genes in cells cultured on the scaffolds under basal and osteogenic conditions. All scaffold formulations were cytocompatible and supported cell adhesion and proliferation. Culture on biomorphic Mg^2+^-doped HA/β-TCP scaffolds promoted the expression of osteogenic marker genes, and this response was further enhanced following osteogenic stimulation. No statistically significant differences among the investigated formulations were detected in cell viability or osteogenic marker gene expression. Overall, these findings demonstrate that biomorphic Mg^2+^-doped HA/β-TCP scaffolds provide a favorable microenvironment for pre-osteoblastic cell growth and early osteogenic transcriptional commitment. The present work also establishes a simplified experimental model for investigating the contribution of magnesium to the early osteogenic transcriptional response of biomorphic calcium phosphate scaffolds, providing a rational basis for the development of next-generation bone graft substitutes.

## 1. Introduction

Critical-sized bone defects remain a major clinical challenge because of their limited intrinsic healing capacity and the high incidence of delayed union and non-unions. Although autologous bone grafting remains the clinical gold standard, its clinical use is constrained by donor-site morbidity, limited tissue availability and increased surgical burden. Consequently, considerable research efforts have been directed towards the development of synthetic bone substitutes capable of promoting bone regeneration within bone tissue engineering (BTE) strategies.

Among synthetic bone graft substitutes, calcium phosphate (CaP) ceramics, particularly hydroxyapatite (HA; Ca_10_(PO_4_)_6_(OH)_2_) and β-tricalcium phosphate (β-TCP; Ca_3_(PO_4_)_2_), represent one of the most extensively investigated classes of biomaterials because of their close chemical similarity to the mineral phase of bone, excellent biocompatibility and well-established osteoconductive properties [1,2]. Biphasic calcium phosphates (BCPs), combining HA and β-TCP in different proportions, are particularly attractive because they integrate the long-term structural stability of HA with the higher resorbability of β-TCP. Consequently, the HA/β-TCP ratio provides an effective means of tailoring scaffold degradation kinetics, mechanical stability and biological performance according to the intended clinical application [3,4].

Besides chemical composition, scaffold architecture plays a pivotal role in regulating the biological response. Pore size, pore interconnectivity and hierarchical organization strongly influence cell colonization, nutrient transport, vascular invasion and tissue ingrowth, thereby contributing to the regenerative performance of three-dimensional scaffolds [5].

Among the available fabrication approaches, biomorphic transformation of natural wood templates has emerged as an innovative strategy for producing calcium phosphate scaffolds that reproduce the hierarchical organization of trabecular bone. In particular, rattan wood represents an excellent template because its naturally interconnected vascular network is preserved throughout the biomorphic conversion process, generating anisotropic porous scaffolds that closely resemble cancellous bone and provide favorable conditions for cell colonization, nutrient diffusion and tissue ingrowth [6,7,8].

In addition to reproducing bone architecture, increasing attention has been devoted to mimicking the chemical complexity of native bone mineral. Unlike stoichiometric hydroxyapatite, biological apatite is a calcium-deficient, poorly crystalline and ion-substituted mineral phase whose physicochemical and biological properties are strongly influenced by both cationic and anionic substitutions. Consequently, controlled ionic substitution has become an established strategy for tailoring the structural, physicochemical and biological characteristics of synthetic calcium phosphate biomaterials [9].

Among the ions naturally present in bone mineral, magnesium has emerged as one of the most extensively investigated dopants because of its central role in skeletal development and mineral metabolism. Magnesium is preferentially incorporated into newly formed bone mineral, where it regulates apatite nucleation, crystal growth and crystallite maturation. In synthetic calcium phosphates, Mg^2+^ incorporation reduces crystallinity, modifies lattice organization and generally enhances material reactivity and dissolution behavior. These physicochemical modifications have been associated with improved cell-material interactions and enhanced early osteogenic responses, making magnesium substitution an attractive strategy for the development of advanced calcium phosphate biomaterials [10,11,12].

Several calcium phosphate biomaterials incorporate magnesium together with other biologically relevant ions, particularly strontium, to better reproduce the compositional complexity of native bone mineral and modulate the physicochemical and biological properties of the material. Clinically available biomorphic scaffolds, such as b.Bone^®^ (GreenBone, Faenza, Italy), exemplify this strategy by combining Mg^2+^ and Sr^2+^ within a hierarchically organized calcium phosphate matrix [13]. However, simultaneous incorporation of multiple dopants generates coupled effects on crystal chemistry, phase composition, dissolution behavior and ionic release, making it difficult to distinguish the individual contribution of each ion to the overall biological response [14,15].

In this context, the present study investigates a series of biomorphic Mg^2+^-doped HA/β-TCP scaffolds in which controlled magnesium incorporation is associated with systematic modulation of the HA/β-TCP phase composition. This experimental approach enables the relationship between Mg^2+^ content, scaffold phase composition and biological response to be evaluated across a defined compositional range, while avoiding the additional complexity introduced by simultaneous Mg^2+^/Sr^2+^ co-doping.

Accordingly, biomorphic Mg^2+^-doped HA/β-TCP scaffolds with different phase compositions were produced through a biomorphic transformation process using rattan wood as a hierarchical structural template. Their biological performance was evaluated using MC3T3-E1 pre-osteoblastic cells by assessing cytocompatibility, cell adhesion and proliferation, together with the expression of representative osteogenic marker genes under basal and osteogenic culture conditions.

## 2. Materials and Methods

### 2.1. Fabrication of Mg-Doped Biomorphic Calcium Phosphate Scaffolds

Two types of biomorphic calcium phosphate scaffolds were investigated in this study: (i) experimental Mg^2+^-doped HA/β-TCP scaffolds with different phase compositions, specifically fabricated for the present study, and (ii) the commercial Mg^2+^/Sr^2+^-containing b.Bone^®^ scaffold (GreenBone, Faenza, Italy), used as a reference material. Both scaffold types were obtained using rattan wood (*Calamus manan*) as a hierarchical structural template through a previously described multistep biomorphic transformation process, with minor modifications [6,7,8]. Cylindrical wood specimens (10 mm diameter) were pyrolyzed at 900 °C under a nitrogen atmosphere (heating/cooling rate: 1 °C min^−1^) to remove the organic phase and obtain carbon templates.

The carbon structures were converted into calcium carbide (CaC_2_) by exposure to calcium vapor generated from metallic calcium granules (Merck KGaA, Darmstadt, Germany) at 1200 °C in a vacuum furnace under argon atmosphere (100 mbar; heating/cooling rate: 2 °C min^−1^). Subsequent oxidation in air at 1100 °C produced calcium oxide (CaO), which was carbonated under wet supercritical CO_2_ (100 atm; CO_2_/H_2_O molar ratio 8–10) at 800 °C to obtain calcium carbonate (CaCO_3_) templates.

Conversion of CaCO_3_ into calcium phosphate was achieved by hydrothermal phosphatization in 3.0 M (NH_4_)_2_HPO_4_ solution (Merck KGaA, Darmstadt, Germany; pH 8.5) at 220 °C under autogenous pressure (~20 bar), producing biomorphic calcium phosphate scaffolds while preserving the hierarchical wood architecture.

Magnesium incorporation was subsequently achieved by immersing the scaffolds for 24 h at 50 °C in buffered (pH 7.0) MgCl_2_ solutions of increasing concentration (0.25, 0.50, 0.70 and 0.75 M), generating four experimental groups with progressively different magnesium contents and HA/β-TCP phase compositions.

Commercial biomorphic b.Bone^®^ scaffolds (GreenBone, Faenza, Italy) were prepared according to [6] by treatment of calcium phosphate templates in an aqueous solution containing 0.5 M MgCl_2_ and 0.5 M SrCl_2_ under identical processing conditions.

All cylindrical scaffolds were sectioned into discs approximately 3–4 mm thick, sterilized by immersion in 70% ethanol for 2 h, and then air-dried under a laminar flow hood for 24 h at room temperature, with no macroscopic changes (e.g., cracking, discoloration) observed, before biological testing.

### 2.2. Physicochemical Characterization

Scaffold phase composition was determined by X-ray diffraction (XRD). Samples were ground into fine powder using a ball mill (Pulverisette 0, Fritsch, Idar-Oberstein, Germany) before analysis.

Diffraction patterns were collected using an Aeris diffractometer (Malvern Panalytical, Almelo, The Netherlands) equipped with CuKα radiation (λ = 1.54178 Å), over the 2θ range 10–80°, using a step size of 0.02° and a counting time of 0.5 s per step.

Quantitative phase analysis was performed by Rietveld refinement using HighScore Plus software (version 4.9; Malvern Panalytical, Almelo, The Netherlands) to determine the relative amounts of hydroxyapatite (HA) and β-tricalcium phosphate (β-TCP). Reference patterns employed for refinement were 06-901-0492 (β-TCP), 96-901-1092 (HA) and 96-901-6707 (calcite).

Magnesium content was quantified by inductively coupled plasma optical emission spectroscopy (ICP-OES; Agilent 5100, Agilent Technologies, Santa Clara, CA, USA). Approximately 20 mg of powdered scaffold was dissolved in 2 mL of 65% nitric acid (Merck KGaA, Darmstadt, Germany), diluted to 100 mL with ultrapure water and analyzed against certified elemental standards (1000 ppm; Merck KGaA). Magnesium concentration was expressed as weight percentage (wt%).

### 2.3. Cell Culture

The murine pre-osteoblastic cell line MC3T3-E1 (ECACC, Salisbury, UK) was cultured in α-MEM without ascorbic acid (Gibco, Life Technologies, Monza, Italy), supplemented with ribonucleosides, deoxyribonucleosides, 2 mM L-glutamine, 1 mM sodium pyruvate, 10% fetal bovine serum (Euroclone, Milan, Italy), 100 U mL^−1^ penicillin and 100 μg mL^−1^ streptomycin.

Cells were maintained at 37 °C in a humidified atmosphere containing 5% CO_2_.

### 2.4. Indirect Cytotoxicity Assay

Indirect cytotoxicity was evaluated according to ISO 10993-5 [16] using conditioned medium prepared from b.Bone^®^ scaffolds. Scaffolds were incubated in complete α-MEM at a ratio of 0.2 g mL^−1^ under gentle agitation for 24 h. The conditioned medium was sterilized through a 0.22 μm filter before use. MC3T3-E1 cells were seeded in 24-well plates at 1.5 × 10^5^ cells cm^−2^ and cultured overnight before replacing the culture medium with scaffold-conditioned medium. Cell metabolic activity was evaluated immediately before and 24 h after treatment using the PrestoBlue^®^ Cell Viability Reagent (Life Technologies). Cell number was estimated from calibration curves generated in parallel experiments. Two independent experiments were performed using three technical replicates each.

### 2.5. Cell Adhesion and Proliferation on Biomorphic Scaffolds

Prior to cell seeding, scaffolds were pre-conditioned in complete culture medium for 72 h to equilibrate the material surface and promote protein adsorption. MC3T3-E1 cells were seeded onto individual scaffolds at a density of 2.5 × 10^5^ cells per scaffold in a 30 μL suspension. Following a 2 h attachment period, 2 mL of complete medium was gently added to each well. To minimize the contribution of any cells not retained by the scaffold, seeded scaffolds were transferred to fresh, clean well plates before each measurement time point. Cell metabolic activity was evaluated after 2, 4 and 8 days using PrestoBlue^®^, according to the manufacturer’s protocol. Cell number was estimated from calibration curves generated in parallel. Cell doubling time (DT) was calculated according to Equation (1):

(1) $$\mathrm{DT}=t\cdot \frac{\ln(2)}{\ln(\frac{C_{f}}{C_{i}})}$$

where ln denotes the natural logarithm, *C_i_* and *C_f_* represent the initial and final cell numbers, respectively, and *t* is the culture time interval.

Cell distribution within the scaffold was evaluated by DAPI staining.

### 2.6. Osteogenic Induction

For osteogenic studies, scaffolds were cultured for six days under standard growth conditions before osteogenic induction. Differentiation medium consisted of complete α-MEM supplemented with 0.2 mM L-ascorbic acid-2-phosphate, 10 mM β-glycerophosphate, and 50 nM melatonin (from day 7 onward). To minimize the contribution of any cells not retained by the scaffold, seeded scaffolds were transferred to fresh, clean wells before the start of osteogenic treatment. Culture medium was replaced three times per week (50% volume replacement), with complete renewal before melatonin supplementation [17].

### 2.7. Quantitative Real-Time PCR

Total RNA was isolated using the NucleoSpin RNA kit (Macherey-Nagel, Düren, Germany). To maximize recovery of cells embedded within the porous scaffold architecture, individual scaffolds were mechanically fragmented immediately before RNA extraction. 500 ng of RNA was reverse-transcribed using the iScript cDNA Synthesis Kit (Bio-Rad Laboratories, Hercules, CA, USA). Quantitative real-time PCR was performed using SsoAdvanced Universal SYBR Green Supermix (Bio-Rad) on a CFX Connect™ Real-Time PCR Detection System under the following cycling conditions: 95 °C for 2 min, 40 cycles of 95 °C for 5 s, 60 °C for 30 s, followed by melting curve analysis. Expression of representative early (Alkaline phosphatase—*ALPL*, Runt-related transcription factor 2—*RUNX2*, Collagen type I alpha 1 chain*—COL1A1*) and late (Osteocalcin—*BGLAP*, Osteonectin*—SPARC*) osteogenic markers was quantified using the 2^−ΔΔCt^ method. Hypoxanthine phosphoribosyltransferase*—HPRT* together with TATA-box binding protein like 1—*TBPL1* served as reference genes.

Primer sequences are reported in Supplementary Table S1.

### 2.8. Statistical Analysis

Data are presented as mean ± standard deviation (SD) or standard error of the mean (SEM), as indicated in the figure legends. Statistical analysis and graphical representations were performed using GraphPad Prism (version 4.0, GraphPad Software, Boston, MA, USA). Cell viability and proliferation data were analyzed using two-way repeated-measures ANOVA followed by Šídák’s multiple comparison test. Gene-expression data obtained from three independent biological replicates were analyzed using two-way ANOVA followed by Tukey’s post hoc test. Differences were considered statistically significant when *p* < 0.05.

## 3. Results

### 3.1. Magnesium-Induced Modulation of Scaffold Phase Composition

The physicochemical characterization demonstrated that increasing magnesium incorporation progressively modified the phase composition of the biomorphic scaffolds. Quantitative X-ray diffraction (XRD) analysis combined with Inductively Coupled Plasma—Optical Emission Spectroscopy (ICP-OES) measurements revealed a direct relationship, within the tested range, between magnesium content and the relative proportion of hydroxyapatite (HA) and β-tricalcium phosphate (β-TCP) (Table 1).

**Figure 1:**
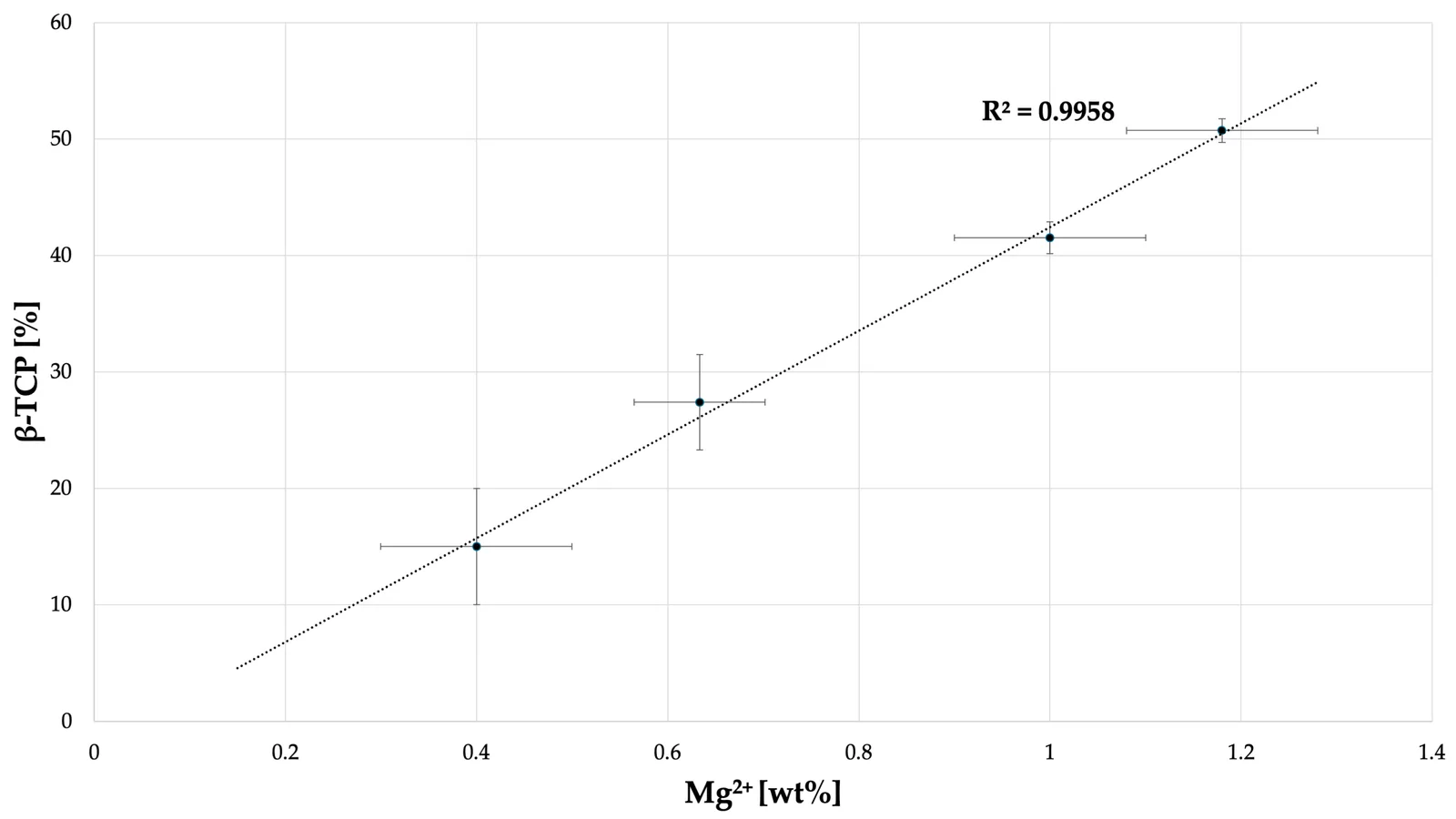
Scatter plot of β-TCP phase content [%] as a function of magnesium content (Mg^2+^ [wt%]) determined by ICP-OES. Each point represents one of the four scaffold formulations (mean ± SD of three scaffolds per group). The dotted line represents the linear regression fit (R^2^ = 0.9958).

The values reported in Table 1 are also presented in a scatter plot, with magnesium content on the x-axis and β-TCP phase percentage on the y-axis (Figure 1).

Scaffolds prepared using the lowest MgCl_2_ concentration (0.25 M) contained 0.4 ± 0.1 wt% magnesium and were composed predominantly of HA (85.0 ± 5.0%), with only 15.0 ± 5.0% β-TCP. Increasing the magnesium concentration during the doping process progressively shifted the phase composition towards higher β-TCP contents. The scaffold prepared with 0.75 M MgCl_2_ reached a magnesium content of 1.2 ± 0.1 wt% and exhibited an almost equivalent proportion of HA (49.3 ± 1.0%) and β-TCP (50.7 ± 1.0%).

**Figure 2:**
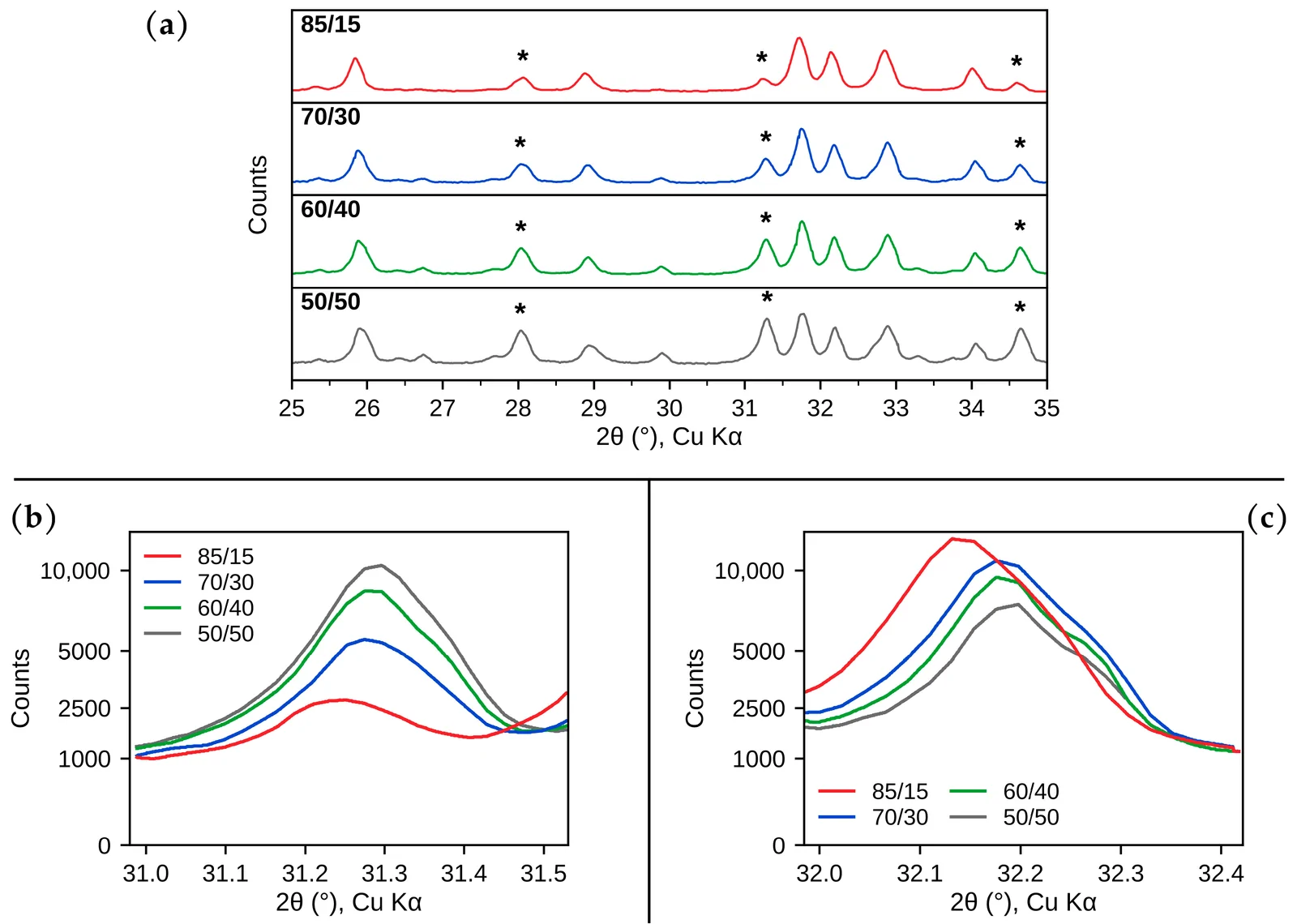
(**a**) Magnified view of the XRD pattern in the 25–35° 2θ range showing β-TCP peaks (marked with *) corresponding to the (2|1|4), (2|0|−10), and (2|2|0) reflections at approximately 28.0°, 31.2° and 34.6°. The other peaks are attributed to hydroxyapatite. (**b**) Magnification of the XRD pattern in the 31.0–31.5° 2θ range showing β-TCP (2|0|−10) peak. (**c**) Magnification of the XRD pattern in the 32.0–32.4° 2θ range showing HA peak corresponding to the (1|1|2) reflection at 32.2°.

The relationship between magnesium content and β-TCP fraction was approximately linear (R^2^ = 0.9958), demonstrating that magnesium incorporation provides an effective means of controlling scaffold phase composition (Figure 1). Based on these results, the four scaffold formulations are hereafter designated according to their HA/β-TCP ratio as 85/15, 70/30, 60/40 and 50/50, corresponding to 0.25, 0.50, 0.70 and 0.75 M MgCl_2_ doping, respectively (Table 1). Representative XRD patterns confirmed the progressive increase in β-TCP with increasing magnesium incorporation (Figure 2). Besides the expected increase in β-TCP peak intensity, both β-TCP and HA diffraction peaks exhibited a gradual shift towards higher 2θ values as magnesium content increased, indicating that magnesium affected not only phase composition but also crystal lattice organization.

### 3.2. Cytocompatibility and Cell Colonization of Biomorphic Scaffolds

The cytocompatibility of the biomorphic scaffolds was initially evaluated by indirect extraction testing according to ISO 10993-5 [16] using conditioned medium prepared from b.Bone^®^ scaffolds.

**Figure 3:**
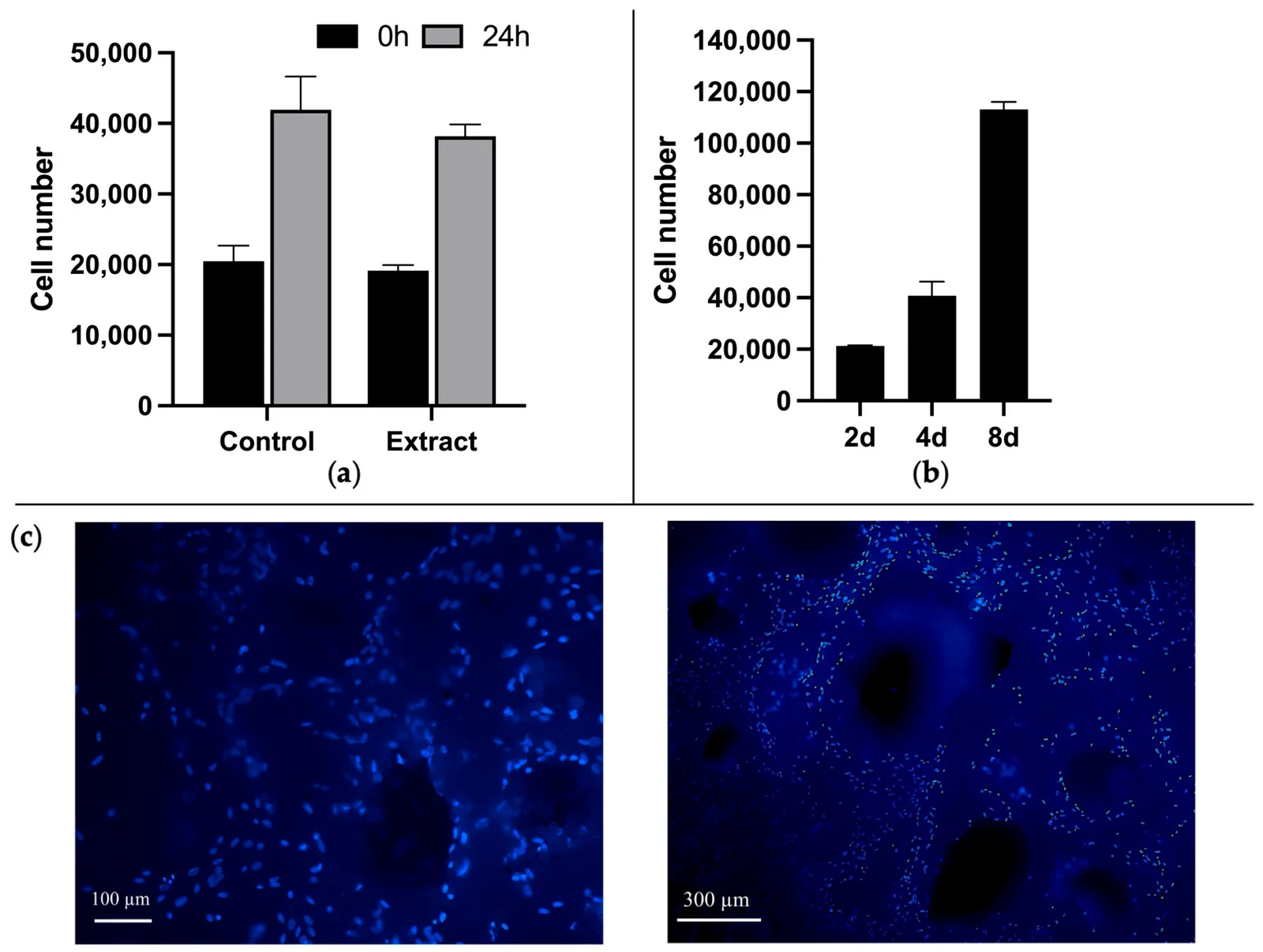
(**a**) Indirect cytotoxicity assay. Viability of MC3T3 cells, cultured in standard media (Control) or in media conditioned with b.Bone^®^ scaffolds (Extract), was assessed at 0 h and 24 h. Data are reported as mean ± standard deviation of one representative experiment performed with three technical replicates. (**b**) Cell viability and proliferation of MC3T3 cells seeded on b.Bone^®^ scaffold, at 2, 4 and 8 days. Data are reported as mean ± standard deviation of one representative experiment performed with three technical replicates. (**c**) DAPI staining (blue) of MC3T3 cells cultured on b.Bone^®^ scaffolds after 6 days, observed at two magnifications: (**Left**) 10×, showing cell distribution and morphology on the scaffold surface; (**Right**) 4×, highlighting the overall pore architecture and cell colonization throughout the structure.

Conditioned medium caused less than a 10% reduction in MC3T3-E1 metabolic activity compared with standard culture medium (Figure 3a), remaining well below the threshold generally considered indicative of cytotoxicity. These findings demonstrate that neither the scaffold composition nor residual products derived from the fabrication process adversely affected cell viability.

Direct culture experiments further confirmed the cytocompatibility of the biomorphic scaffolds. Following seeding onto b.Bone^®^, MC3T3-E1 cells remained viable throughout the observation period and displayed a progressive increase in metabolic activity between days 2 and 8 (Figure 3b). Cell proliferation was more pronounced during the first culture interval (days 2–4), corresponding to a doubling time of approximately 51 h, whereas proliferation slowed slightly during the subsequent interval (days 4–8), consistent with progressive colonization of the three-dimensional scaffold.

Fluorescence microscopy following DAPI staining demonstrated extensive cell colonization of the scaffold (Figure 3c). Cells were observed both on the scaffold surface and within the interconnected pore network, confirming that the biomorphic architecture supports cell infiltration throughout the three-dimensional structure.

Based on these observations, the early culture interval (2–4 days) was selected for subsequent comparisons among the different Mg^2+^-doped scaffold formulations.

### 3.3. Early Cell Proliferation Under Magnesium-Mediated Phase Composition Modulation

The influence of scaffold composition on early cellular behavior was evaluated by culturing MC3T3-E1 cells on scaffolds exhibiting different HA/β-TCP phase ratios.

**Figure 4:**
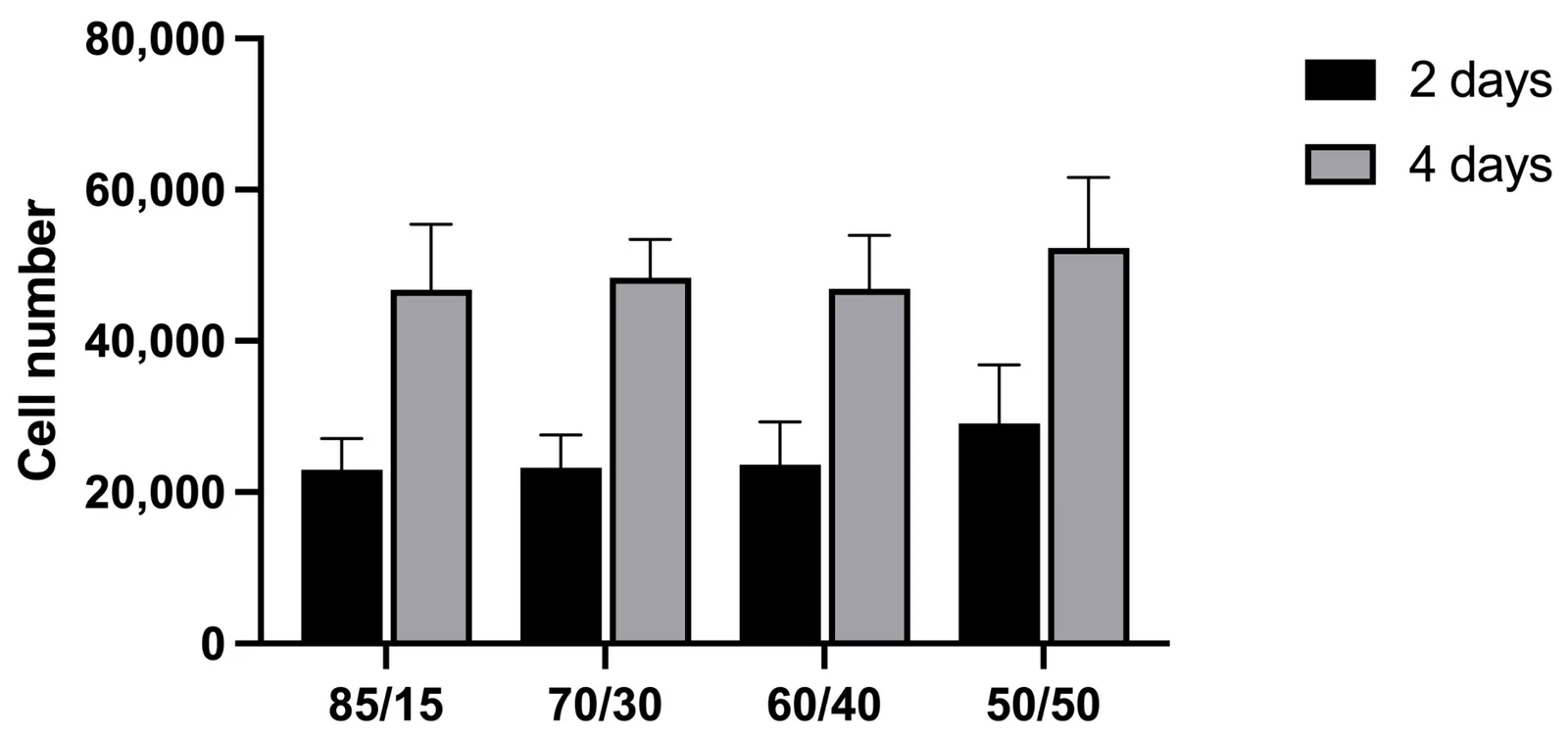
Viability and proliferation of MC3T3 cells seeded on Mg-doped scaffolds at 2 and 4 days. (85/15, 70/30, 60/40, 50/50 HA/β-TCP; see Table 1 for corresponding MgCl_2_ concentrations). Data are reported as mean ± SEM of 8 scaffolds from three independent experiments. All scaffolds showed a significant increase in metabolic activity from day 2 to day 4 (*p* < 0.05; see Table S3).

All scaffold formulations supported comparable levels of cell viability and proliferation throughout the first four days of culture (Figure 4). Cell numbers increased from approximately 2.3–2.9 × 10^4^ cells at day 2 to 4.7–5.2 × 10^4^ cells at day 4, corresponding to an approximately two-fold increase irrespective of scaffold composition.

No statistically significant differences were detected among the four scaffold groups (*p* > 0.05), indicating that the progressive increase in magnesium content and the associated changes in HA/β-TCP phase composition did not affect early cell attachment or proliferation.

Collectively, these findings demonstrate that magnesium-mediated modulation of scaffold phase composition preserves the cytocompatibility of the biomorphic calcium phosphate scaffolds over the investigated compositional range.

### 3.4. Early Osteogenic Transcriptional Response of Biomorphic Scaffolds

#### 3.4.1. Exploratory Time-Course of Osteogenic Marker Expression on b.Bone^®^

**Figure 5:**
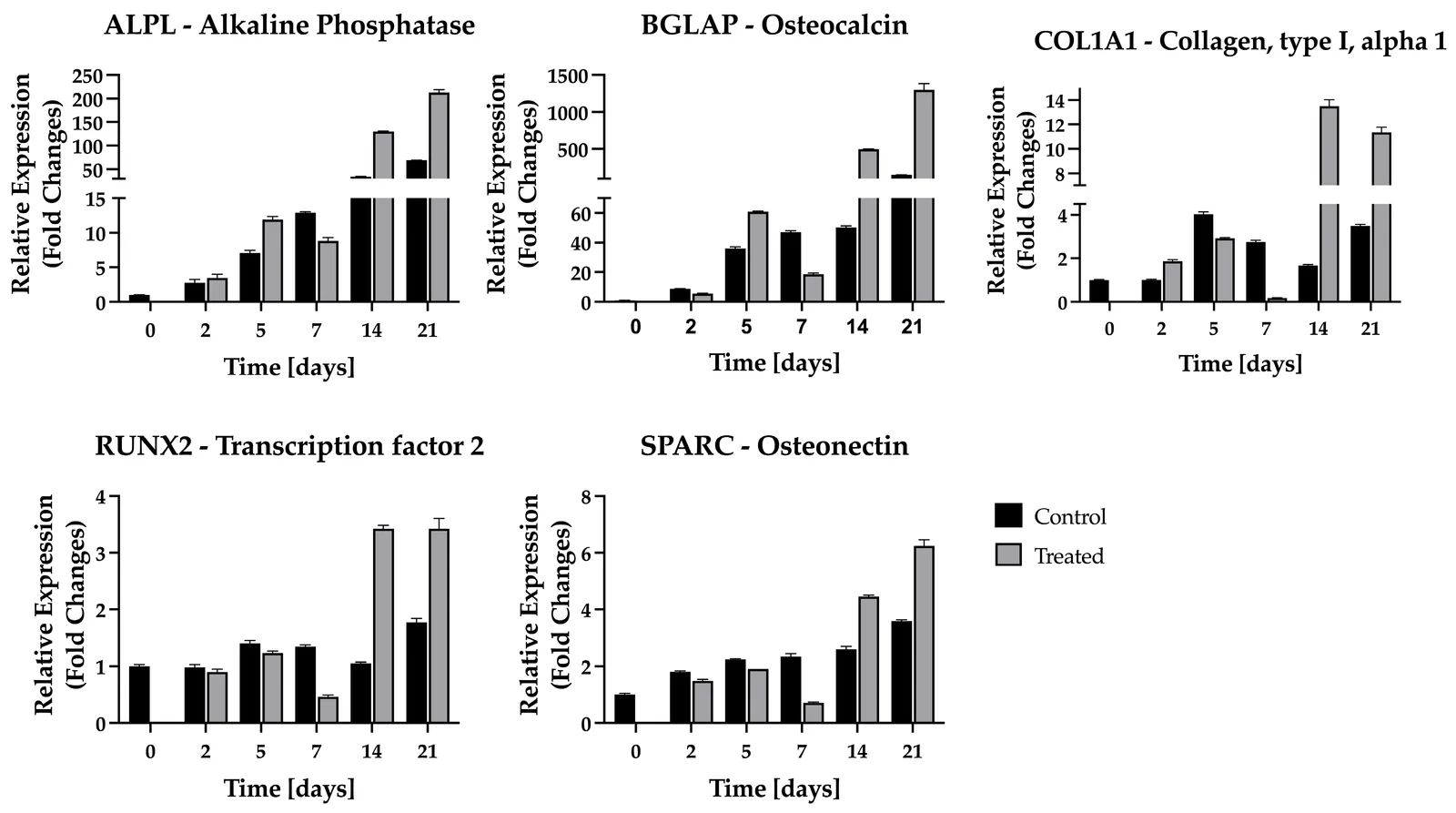
Exploratory time-course analysis of osteogenic marker gene expression in MC3T3-E1 cells cultured on b.Bone^®^ under basal and osteogenic conditions. Expression was evaluated at days 0, 2, 5, 7, 14 and 21 and normalized to the day-0 reference condition. Data were derived from a single biological time-course experiment, with two technical replicates per time point, and are presented descriptively; no inferential statistical analysis was performed. *HPRT* and *TBPL1* were used as reference genes and data are reported as mean ± SEM.

An exploratory time-course experiment was initially performed to characterize the temporal profile of osteogenic marker gene expression in MC3T3-E1 cells cultured on b.Bone^®^ and to identify an informative endpoint for the subsequent comparative analysis of the Mg^2+^-doped scaffold formulations. Cells were cultured for up to 21 days under basal or osteogenic conditions, and expression of the early osteogenic markers *ALPL*, *RUNX2* and *COL1A1* and the late markers *BGLAP* and *SPARC* was evaluated at days 0, 2, 5, 7, 14 and 21 (Figure 5).

The exploratory expression profiles showed a time-dependent increase in both early and late osteogenic markers under basal and osteogenic culture conditions. Osteogenic supplementation was associated with a more pronounced increase in marker expression, particularly from day 14 onwards. Because this experiment consisted of a single biological time course, these observations were used only to guide endpoint selection and were not subjected to inferential statistical analysis.

Based on this exploratory profile, day 14 was selected as a practical and biologically informative endpoint for the subsequent comparison of the different Mg^2+^-doped scaffold formulations. The comparative day-14 experiment was subsequently performed using three independent biological replicates and analyzed statistically. Overall, this exploratory time-course suggested that the biomorphic scaffold provides a favorable environment for the expression of osteogenic marker genes under basal conditions, with a more pronounced transcriptional response following osteogenic supplementation. As the expression profiles showed a clearly detectable response by day 14, this time point was selected as a practical and biologically informative endpoint for the subsequent replicated comparison of the Mg^2+^-doped scaffold formulations, while avoiding extension of the culture period to 21 days.

#### 3.4.2. Early Osteogenic Transcriptional Response of Mg^2+^-Doped Scaffolds

**Figure 6:**
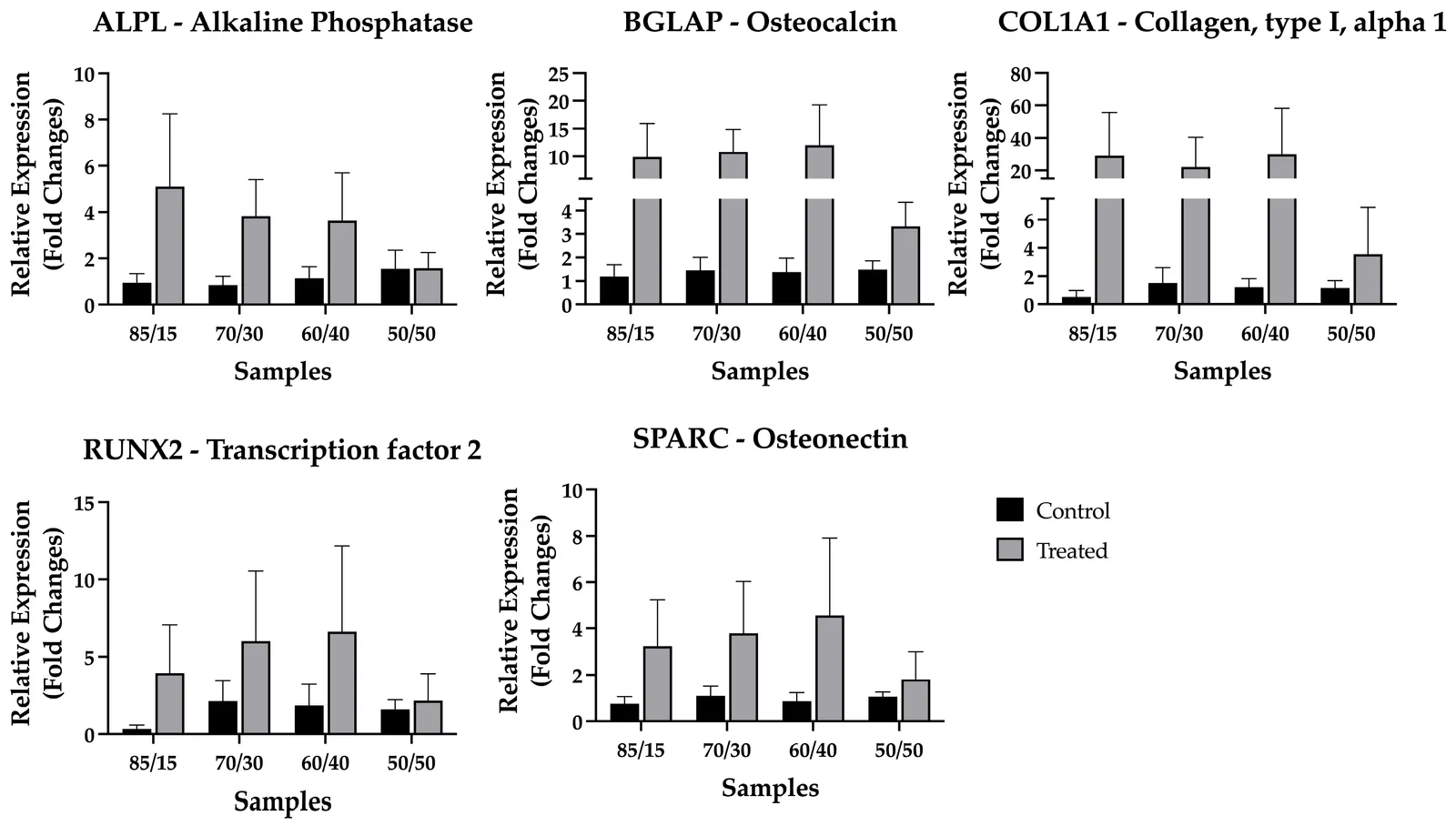
Osteogenic gene marker expression of MC3T3 cells seeded on Mg^2+^-doped scaffolds (85/15, 70/30, 60/40, 50/50 HA/β-TCP; see Table 1 for corresponding MgCl_2_ concentrations). qPCR analysis of osteogenic markers (*ALPL*, *BGLAP*, *COL1A1*, *RUNX2*, *SPARC*) was performed on cells cultured with proliferation media (Control) and osteogenic cocktail media (Treated) at day 14. *HPRT* and *TBPL1* were used as reference genes (2^−ΔΔCt^ method). Fold changes were calculated against b.Bone^®^ (Control). All scaffolds showed a significant increase in expression under treated conditions (*p* < 0.05, see Table S2). All data are reported as mean ± SEM of three biological replicates.

Based on the temporal expression profile observed on the reference biomorphic scaffold, osteogenic marker gene expression was subsequently evaluated after 14 days of culture in both b.Bone^®^ and the Mg^2+^-doped scaffold formulations under basal and osteogenic conditions (Figure 6). Gene expression levels were normalized to those measured in b.Bone^®^ at day 14.

As observed in the time-course experiment, osteogenic stimulation consistently enhanced the expression of all investigated marker genes irrespective of scaffold composition.

All Mg^2+^-doped scaffold formulations supported expression of both early (*ALPL*, *RUNX2*, *COL1A1*) and late (*BGLAP*, *SPARC*) osteogenic marker genes. Under osteogenic conditions, maximum fold changes reached 5.1 for *ALPL*, 6.6 for *RUNX2*, 29.9 for *COL1A1*, 12.0 for *BGLAP*, and 4.5 for *SPARC*.

Although moderate variability was observed among individual osteogenic markers, no statistically significant magnesium-dependent effect was detected on osteogenic marker expression within the tested sample size (*n* = 3 biological replicates per group). Overall, all Mg^2+^-doped scaffold formulations supported early osteogenic marker expression and remained responsive to osteogenic stimulation, with no consistent association between scaffold formulation and gene expression detected under the experimental conditions investigated.

Collectively, these findings demonstrate that magnesium provides an effective means of modulating scaffold phase composition, with all investigated Mg^2+^-doped formulations supporting cytocompatibility and early osteogenic marker expression under the experimental conditions examined.

## 4. Discussion

The present study demonstrates that controlled magnesium incorporation provides an effective strategy for modulating the physicochemical characteristics of biomorphic calcium phosphate scaffolds while preserving cytocompatibility and supporting the early osteogenic transcriptional response of pre-osteoblastic cells. By investigating magnesium as the sole intentionally introduced ionic dopant, this work also establishes a simplified experimental model for exploring structure–property–function relationships in biomorphic calcium phosphate scaffolds, reducing the complexity associated with clinically available multi-substituted biomaterials.

### 4.1. Magnesium-Driven Coupled Modifications of Scaffold Physicochemistry

One of the most relevant observations emerging from this study is the close relationship between magnesium incorporation and scaffold phase composition. Increasing magnesium progressively shifted the balance from HA-rich formulations towards increasingly biphasic HA/β-TCP compositions, while simultaneously modifying crystal lattice organization, as demonstrated by systematic shifts in the XRD reflections.

Besides promoting the formation of β-TCP, magnesium induced measurable peak shifts in both the β-TCP and HA phases. Although lattice parameters were not determined in the present study, these shifts suggest that Mg^2+^ was incorporated into the crystal lattices of both phases rather than being exclusively associated with β-TCP; Rietveld-refined lattice parameters would provide a more rigorous quantitative confirmation of this partitioning and represent a target for future characterization.

These findings agree with previous reports showing that Mg^2+^ destabilizes hydroxyapatite crystallization owing to its smaller ionic radius and distinct coordination environment, favoring the formation of more soluble calcium phosphate phases and reducing apatite crystallinity [11,12]. Magnesium is widely recognized as a β-phase stabilizer, and its preferential partitioning into β-TCP during the formation of biphasic calcium phosphates has been extensively reported [18]. Consistent with this behavior, the increasing β-TCP fraction observed here confirms the well-established β-phase-stabilizing effect of magnesium [19]. However, the simultaneous shift in the HA reflections suggests that magnesium is also incorporated, at least to some extent, into the hydroxyapatite lattice. To the best of our knowledge, this phenomenon has been only rarely described in biphasic calcium phosphates and may represent a distinctive feature of the GreenBone biomorphic transformation process.

This observation is particularly relevant because hydroxyapatite is intrinsically less soluble and more slowly resorbed than β-TCP. Therefore, partial magnesium incorporation into the HA lattice may provide an additional mechanism to tailor the dissolution and resorption behavior of the HA phase itself, beyond simply increasing the β-TCP content [20]. This could contribute to a finer control of scaffold remodeling while preserving the biomimetic architecture of the material.

### 4.2. Contribution of Biomorphic Architecture and Phase Modulation to Cytocompatibility

Despite substantial modifications of scaffold physicochemistry, all formulations remained highly cytocompatible. Neither extraction tests nor direct culture experiments indicated adverse effects on MC3T3-E1 viability, confirming that the biomorphic fabrication process produces biologically compatible scaffolds over the investigated compositional range.

The preserved hierarchical pore architecture inherited from the rattan template is likely to contribute substantially to this behavior. Previous studies have shown that anisotropic interconnected porosity promotes efficient cell colonization, nutrient transport and tissue ingrowth, thereby supporting the biological performance of biomorphic calcium phosphate scaffolds [5,6,8,21].

Equally important, modulation of the HA/β-TCP ratio did not compromise early cell proliferation. Although the scaffold compositions differed markedly in both magnesium content and phase composition, no significant differences in metabolic activity were detected. These findings indicate that magnesium-mediated phase modulation can be exploited to tailor scaffold physicochemistry without adversely affecting the earliest stages of cell-material interaction.

### 4.3. Preservation of the Early Osteogenic Transcriptional Response Under Magnesium-Mediated Phase Modulation

An exploratory time-course analysis performed on the reference b.Bone^®^ scaffold indicated a progressive increase in osteogenic marker expression during culture, with a more pronounced response to osteogenic supplementation from day 14 onwards. Because this preliminary experiment consisted of a single biological time course, it was not intended to provide quantitative or statistical evidence of temporal differences in gene expression. Rather, it was used to identify a biologically informative endpoint for the subsequent replicated comparison among the Mg^2+^-doped scaffold formulations. Based on the observed expression profiles, day 14 was selected for this comparative analysis.

At day 14, analysis of three independent biological replicates showed comparable expression across the Mg^2+^-doped scaffold formulations, with no statistically significant effect of scaffold formulation for any of the genes analyzed (Table S2). Osteogenic supplementation consistently enhanced the transcriptional response across the investigated formulations. Despite marked differences in Mg content and HA/β-TCP phase composition, no consistent Mg-dependent or phase composition-dependent trend in osteogenic marker expression was observed. These findings indicate that the investigated range of Mg-mediated phase modulation is compatible with the maintenance of an early osteogenic transcriptional response.

These observations are consistent with previous reports showing an association between intracellular magnesium homeostasis, osteogenic commitment and extracellular matrix mineralization in three-dimensional culture systems [22]. Although intracellular Mg dynamics were not investigated in the present study, the comparable osteogenic transcriptional profiles observed across the different scaffold formulations indicate that Mg-mediated changes in HA/β-TCP phase composition occur within a compositional range that remains favorable to the osteogenic response of pre-osteoblastic cells.

Importantly, the present study focused on transcriptional activation rather than functional osteogenic differentiation. The findings should therefore be interpreted as evidence of a maintained early osteogenic transcriptional response across the investigated Mg^2+^-doped scaffold formulations. Additional biochemical and functional analyses will be required to determine whether these transcriptional responses translate into functional osteoblastic maturation.

### 4.4. Study Limitations and Future Perspectives

The present study has some limitations that should be considered when interpreting the biological findings. A primary limitation is the absence of a Mg-free biomorphic scaffold within the experimental series. Consequently, the present study does not allow the biological contribution of magnesium itself to be distinguished from the concomitant changes in HA/β-TCP phase composition induced by Mg incorporation. The results should therefore be interpreted in terms of the biological response associated with Mg-mediated modulation of scaffold phase composition rather than as evidence of an independent biological effect of Mg^2+^. Future studies including Mg-free controls and, where technically feasible, formulations in which Mg content and HA/β-TCP phase ratio are independently varied will be required to disentangle the respective contributions of these parameters.

A further limitation is that the biological characterization was primarily based on transcriptional analyses performed using a single murine pre-osteoblastic cell line. Although osteogenic marker gene expression provides valuable information on early osteogenic commitment, it does not by itself demonstrate functional osteoblastic differentiation. Future studies should therefore complement the present molecular observations with biochemical and functional endpoints, including alkaline phosphatase activity, collagen deposition and extracellular matrix mineralization, to determine whether the observed transcriptional responses translate into functional osteoblastic maturation.

The influence of dynamic culture conditions also deserves further investigation. Previous studies have demonstrated that perfusion bioreactors can enhance nutrient transport, cell infiltration and osteogenic commitment in three-dimensional tissue-engineered constructs [23,24]. Evaluation of Mg^2+^-doped biomorphic scaffolds under controlled perfusion conditions may therefore provide a more physiologically relevant assessment of cell–scaffold interactions and reveal biological differences among scaffold formulations that are not detectable under static culture conditions.

Finally, recent advances in non-destructive monitoring technologies for three-dimensional cultures provide opportunities for longitudinal assessment of scaffold colonization and osteoblastic maturation without sacrificing the constructs [25]. Integration of these approaches with biomorphic scaffold platforms may contribute to a more comprehensive understanding of the relationships among Mg^2+^ incorporation, HA/β-TCP phase composition, cellular behavior and bone tissue regeneration.

## 5. Conclusions

The present study identifies controlled magnesium incorporation as an effective processing parameter for tuning the HA/β-TCP phase composition of biomorphic calcium phosphate scaffolds while maintaining cytocompatibility and a favorable early osteogenic transcriptional response of pre-osteoblastic cells across the investigated compositional range. All scaffold formulations supported comparable cell adhesion, proliferation and osteogenic transcriptional responses, indicating that variations in magnesium content and the associated HA/β-TCP phase composition within this range were not major determinants of the observed cellular response.

These findings suggest that the biological performance of the scaffolds is likely governed by the combined contribution of multiple material features, including their hierarchically organized three-dimensional architecture, nanocrystalline structure and biomimetic chemical composition, whose beneficial effects have previously been demonstrated in comparison with conventional sintered, non-hierarchical calcium phosphate scaffolds [26]. Within this context, Mg-mediated phase modulation provides a useful strategy for tailoring the composition of biomorphic HA/β-TCP scaffolds while preserving favorable biological properties, supporting further optimization of these materials as advanced bone substitutes for bone tissue engineering applications.

## Figures and Tables

**Table 1:** Phase composition (HA [%] and β-TCP [%], XRD) and magnesium quantity (Mg^2+^ [wt%], ICP-OES) of the synthesized scaffolds. Magnesium quantity introduced in the doping phase solution is reported as Mg^2+^[M]. Data are reported as mean ± standard deviation of three different scaffolds per group. Trace amounts of calcite, within the margin of error of the Rietveld refinement, were also detected but are not reported as they were considered negligible.

Mg^2+^ [M]	Mg^2+^ [wt%]	HA [%]	β-TCP [%]
0.25	0.4 ± 0.1	85.0 ± 5.0	15.0 ± 5.0
0.5	0.6 ± 0.1	72.6 ± 4.1	27.4 ± 4.1
0.7	1.0 ± 0.1	58.5 ± 1.4	41.5 ± 1.4
0.75	1.2 ± 0.1	49.3 ± 1.0	50.7 ± 1.0

## Data Availability

The data presented in this study are openly available in Zenodo at https://doi.org/10.5281/zenodo.21699936. Data related to XRD and ICP-OES analyses are not publicly available due to proprietary restrictions imposed by GreenBone Ortho S.p.A. and can be made available from the corresponding author upon reasonable request, subject to authorization from GreenBone Ortho S.p.A.

## Supplementary Materials

The following supporting information can be downloaded at: https://www.mdpi.com/article/10.3390/cells15201885/s1, Table S1: Primer sequences; Table S2: Two-way ANOVA *p*-values for the ’scaffold formulation’ factor (four Mg^2+^-doped HA/β-TCP groups) on osteogenic marker gene expression (related to Figure 6; *n* = 3 biological replicates per group); Table S3: Doubling time and adjusted *p*-values (Bonferroni-Šídák) for the Day 2 vs. Day 4 comparison of cell proliferation for each Mg^2+^-doped HA/β-TCP scaffold formulation (related to Figure 4; *n* = 3 biological replicates per group). Doubling time was calculated according to Equation (1), consistent with the value reported for b.Bone^®^ (~51 h) in Section 3.2.
