# Supplementary material for: Early Osteogenic Transcriptional Response of Pre-Osteoblastic Cells on Biomorphic Mg2+-Doped Biphasic Calcium Phosphate Scaffolds
Source: Cells. 2026 Oct 15;15(20):1885. doi: 10.3390/cells15201885 (PMC13702861; doi:10.3390/cells15201885)
Supplement: Supplementary file 1 [file cells-15-01885-s001.zip › cells-4502524-supplementary.pdf]

Supplementary Materials

Gene		Sequence 5'-3'
ALPL	Forward	ATTCCCACTATGTCTGGAAC
	Reverse	CTCAAAGAGACCTAAGAGGTAG
BGLAP	Forward	ACCATGAGGACCATCTTTC
	Reverse	GGACATGAAGGCTTTGTC
B2M	Forward	GTATGCTATCCAGAAAACCC
	Reverse	CTGAAGGACATATCTGACATC
COL1A1	Forward	CGTATCACCAAACTCAGAAG
	Reverse	GAAGCAAAGTTTCCTCCAAG
HPRT	Forward	AGGGATTTGAATCACGTTG
	Reverse	TTTACTGGCAACATCAACAG
RUNX2	Forward	ACAAGGACAGAGTCAGATTAC
	Reverse	CAGTGTCATCATCTGAAATACG
SPARC	Forward	CGAGACTTTGAGAAGAACTAC
	Reverse	GGACAGGTACCCATCAATAG
TBPL1	Forward	GAAACTAGGTTTTCAGGTCATC
	Reverse	GAAGTTCAGGTTCATAGCTG

Supplementary Table S1: Primer sequences

Gene	p-value
ALPL	0.814
RUNX2	0.783
COL1A1	0.814
BGLAP	0.654
SPARC	0.879

Supplementary Table S2: Two-way ANOVA p-values for the 'scaffold formulation' factor (four Mg2+-doped HA/β-TCP groups) on osteogenic marker gene expression (related to Figure 6; n = 3 biological replicates per group).

Note: Tukey's post hoc pairwise comparisons among the four scaffold formulations (within each culture condition) were also non-significant for all genes (adjusted p > 0.65 in all cases).

Scaffold formulation (HA/β-TCP)	Doubling time (h)	Day 2 vs Day 4, adjusted p-value
85/15	46.8	9.79×10^{-6}
70/30	45.4	4.32×10^{-6}
60/40	48.6	1.39×10^{-5}
50/50	56.7	1.42×10^{-5}

Supplementary Table S3: Doubling time and adjusted p-values (Bonferroni-Šídák) for the Day 2 vs. Day 4 comparison of cell proliferation for each Mg^{2+} doped HA/β-TCP scaffold formulation (related to Figure 4; n = 3 biological replicates per group). Doubling time was calculated according to Equation (1), consistent with the value reported for b.Bone® (~51 h) in Section 3.2.

Note: Two-way repeated-measures ANOVA confirmed a highly significant effect of culture time ($p = 1.8 \times 10^{-11}$), whereas the effect of scaffold formulation was not significant ($p = 0.91$), consistent with comparable proliferation across all tested formulations.
